# Inter-institutional pathology consultations for breast cancer: impact on clinical oncology therapy recommendations

**DOI:** 10.3747/co.v17i1.461

**Published:** 2010-02

**Authors:** J.A. Price, E. Grunfeld, P.J. Barnes, D.E. Rheaume, D. Rayson

**Affiliations:** * Division of Medical Oncology, Department of Oncology, Cross Cancer Institute, Edmonton, AB; † Department of Family and Community Medicine, University of Toronto, Toronto, ON; ‡ Department of Pathology, QEII Health Sciences Centre, Halifax, NS; § Department of Radiation Oncology, QEII Health Sciences Centre, Halifax, NS; || Division of Medical Oncology, QEII Health Sciences Centre, Halifax, NS

**Keywords:** Breast cancer, surgical pathology, pathology review, quality assurance

## Abstract

**Background:**

Despite recommendations favouring review of cancer pathology specimens for patients being treated at an institution other than the one that produced the initial pathology report, data regarding discordance rates and their potential clinical impact remain limited, particularly for breast cancer. At the QEII Health Sciences Centre in Halifax, Nova Scotia, it was routine practice to review histopathology when patients referred for adjuvant therapy had undergone their breast cancer surgery and pathology reporting at another institution. The aim of the present study was to determine the rate and clinical impact of discordance in inter-institutional pathology consultations for breast cancer in Nova Scotia.

**Methods:**

We conducted a retrospective review of 100 randomly selected inter-institutional pathology consultations for breast cancer patients referred to the QEII in 2004. Cases were categorized as having either no discordance, discordance with no clinical impact, or discordance with potential for clinical impact. Cases with potential clinical impact were independently reviewed by 2 medical oncologists and 2 radiation oncologists, and the discordances were rated as having high, medium, or no clinical impact.

**Results:**

The study cohort consisted of 93 cases that met the inclusion criteria. Of these 93 cases, 6 had no discordance, 7 had discordance with no clinical impact, and 80 had discordance with potential for clinical impact. Overall, 10 cases (11%) were rated as having either high or medium clinical impact, with agreement on the clinical impact ratings by oncologist reviewers in the same specialty. The remaining cases had either no clinical impact or disagreement on the clinical impact rating.

**Conclusions:**

Inter-institutional pathology consultations for breast cancer in Nova Scotia identified discordant findings with potential clinical impact as determined by oncologist reviewers. Further evaluation of inter-institutional pathology consultations and the impact on clinical decision-making is warranted.

## 1. INTRODUCTION

Breast cancer is the most common noncutaneous cancer arising in Canadian women. New breast cancer cases in Canada were estimated to reach 22,600 in 2009, with 5400 deaths from the disease. In Nova Scotia, new diagnoses were estimated to reach 690, with 190 breast cancer-related deaths 1.

Nova Scotia has two tertiary oncology referral centres, based in Halifax and Sydney. However, approximately half of new breast cancer surgeries in the province are performed outside the surgical catchment areas of those two regions 2. Pathology for patients who have breast cancer surgery at hospitals throughout mainland Nova Scotia (excluding Cape Breton) is reported at the local institution, and most patients are then referred to the QEII Health Sciences Centre in Halifax for adjuvant medical and radiation oncology assessment.

Until 2005, it had been routine practice for the histopathology accompanying each patient referral to be systematically reviewed by one of a team of four QEII pathologists with interest and expertise in breast pathology. This review is called an inter-institutional pathology consultation. These consultations are a component of quality assurance in pathology and are distinct from direct pathologist-to-pathologist consultations for second opinions on challenging cases. Although this quality assurance procedure was routine practice at our centre, it was never systematically evaluated. Specifically, the rate of discordance between the pathology reports and the clinical impact of the pathology reviews were unknown. In recent years, complete pathology review of external cases has been reduced because of workload constraints.

Previous studies of inter-institutional pathology consultations for a cancer diagnosis have reported overall discordance rates ranging from 1.4% to 9.0% 3–7. Discordance rates for breast cancer pathology have ranged from 1.4% to 6.3% 3–5, and breast cancer–specific pathology reviews performed as part of a dedicated multidisciplinary tumour board have observed a 4%–29% discordance rate 8,9. In most of these series, information regarding the specific discordant elements and the potential clinical impact of the discordant findings is limited.

The aim of the present study was to determine the rate, types, and potential clinical impact of discordance in inter-institutional pathology consultations for breast cancer in Nova Scotia.

## 2. METHODS

**FIGURE 1:**
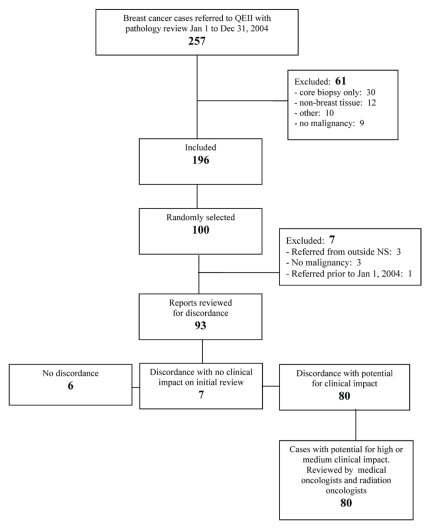
Identification of cases for inclusion.

We conducted a retrospective review of 100 randomly selected inter-institutional pathology consultations for patients with breast cancer referred to the QEII Health Sciences Centre during January 1–December 31, 2004. The computerized anatomic pathology files (Cerner Classic: Cerner Corporation, Kansas City, MO, U.S.A.) were used to identify all breast cancer inter-institutional pathology consultations performed in 2004 and to generate a summary of the consultant’s findings for each case. These summaries were reviewed independently by two investigators (JAP, PJB) to determine eligibility. Figure 1 shows total cases and reasons for exclusion. In cases of uncertainty, the reports were discussed by the two investigators, and a consensus was reached. From among the eligible cases, 100 were randomly selected with the use of a computer-generated random-number sequence. The original pathologist’s reports and the consultant’s reports for these 100 cases were obtained from the QEII Department of Pathology and were reviewed to identify any further cases for exclusion. All patient identifiers were removed from the reports.

For eligible cases, key data elements (Table I) were abstracted from the original and consultation pathology reports based on the breast cancer synoptic reporting format currently in use in Nova Scotia. Data abstraction was performed by a single unblinded investigator (JAP). Based on pre-specified criteria, cases were categorized according to degree of discordance:

- No discordance
- Discordance with no clinical impact (Table II presents the specific discordant criteria considered to have no clinical impact.)
- Discordance with potential for clinical impact

For each case having discordance with potential clinical impact, a summary was prepared of abstracted data from the original and consultation reports. The identity of the summarized report as either original or consultation was indicated. These summary tables were provided to 2 medical oncologists and 2 radiation oncologists with breast cancer subspecialty interest and expertise who, blinded to the other reviewers, assessed the summaries and classified each discordant case as having either high, medium, or no clinical impact. “High clinical impact” was defined as the potential to lead to a change in the intent of treatment or in the treatment modality recommended. “Medium clinical impact” was defined as a potential to change the type or duration of treatment (or both) within a modality or to change the emphasis placed on a recommended modality. Radiotherapy, chemotherapy, hormonal therapy, and additional surgery were considered separate modalities. For cases classified as having discordance with high or medium clinical impact, oncologist reviewers were asked to specifically describe the potential therapeutic implications of the discordance.

Descriptive statistics were used to depict the proportion of cases with clinical impact. Kappa statistics were used to compare clinical impact ratings between oncologists within the same discipline. Approval for the study was obtained from the Capital Health Research Ethics Board.

## 3. RESULTS

We identified 257 cases as potential candidates for inclusion. Of the potential cases, 61 were excluded for the reasons presented in Figure 1. Of the remaining 196 cases, 100 were randomly selected for the study. After the complete reports had been reviewed, 7 more were subsequently excluded (Figure 1), leaving 93 cases in the study cohort.

For the included cases, the original pathology was reported by 10 pathologists at 5 separate centres. In 30 cases, the original pathology was performed by the same pathologist, and 39 reports came from a single institution. Consultations at the QEII were performed by 1 of the 4 pathologists with breast pathology interest and expertise, with 2 of those 4 pathologists accounting for 68 and 22 of the consultations. Table III shows the clinicopathologic characteristics from the original pathology reports for the entire cohort.

After review of the original and consultation pathology reports, 6 of the 93 cases were assessed to have no discordance; 7, to have discordance with no clinical impact as determined by the pre-specified criteria (Table II); and 80, to have discordance with potential for clinical impact. These latter 80 cases were then reviewed independently by 2 medical oncologists and 2 radiation oncologists with subspecialty expertise in breast cancer.

**FIGURE 2:**
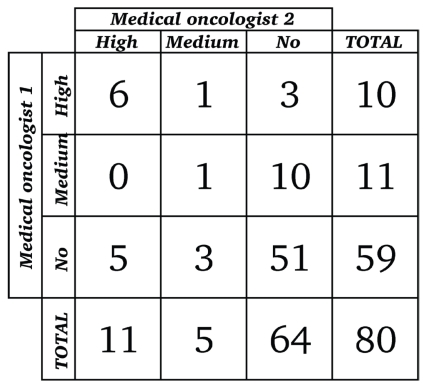
Rating of 80 cases with potential for clinical impact (medical oncologist 1 by medical oncologist 2). Weighted kappa (95% confidence limits): 0.36 (0.12, 0.60). (Kappa < 0.20 = poor agreement; 0.20–0.40 = fair agreement; 0.40–0.60 =moderate agreement; 0.60–0.80 = good agreement; 0.80–1.00 = very good agreement.)

**FIGURE 3:**
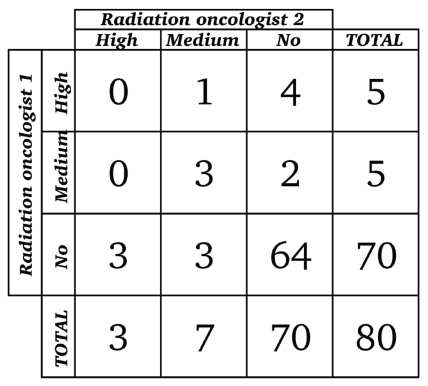
Rating of 80 cases with potential for clinical impact (radiation oncologist 1 by radiation oncologist 2). Weighted kappa (95% confidence limits): 0.20 (−0.03, 0.44). (Kappa < 0.20 = poor agreement; 0.20–0.40 = fair agreement; 0.40–0.60 =moderate agreement; 0.60–0.80 = good agreement; 0.80–1.00 = very good agreement.)

Figures 2 and 3 present the results of the oncologists’ categorization of discordant cases with potential clinical impact. Opinions from the 2 medical oncologists regarding clinical impact agreed for 58 of the 80 cases reviewed: both medical oncologists rated 51 discordances as having no clinical impact, 6 as having high clinical impact, and 1 as having medium clinical impact (Figure 2). Opinions from the 2 radiation oncologists regarding clinical impact agreed for 67 of the 80 cases reviewed: both radiation oncologists rated 64 as having no clinical impact and 3 as having medium clinical impact (Figure 3). Overall, 10 cases (11%) in the study cohort met our criteria for either high or medium clinical impact, with agreement between the oncologist reviewers in each oncology specialty.

For 22 cases, the medical oncologists disagreed on clinical impact. In 21 cases, either high or medium clinical impact was assessed by one reviewer, but the other reviewer assessed no clinical impact; in 1 case, high impact was assessed by one medical oncologist and medium impact by the other (Figure 2). The weighted kappa statistic for agreement between the medical oncologists was 0.36 [95% confidence interval (ci): 0.12 to 0.60].

For 13 cases, the radiation oncologists disagreed on clinical impact. In 12 cases, either high or medium clinical impact was assessed by one reviewer, but the other reviewer assessed no clinical impact; in 1 case, high impact was assessed by one radiation oncologist and medium impact by the other (Figure 3). The weighted kappa statistic for agreement between the radiation oncologists was 0.20 (95% ci: −0.03 to 0.44).

Table IV details the main reasons for discordance in the 6 discordant cases rated as having high clinical impact by both medical oncologists. These cases included a change from *in situ* to microinvasive or invasive disease (2 cases), change in hormone receptor status (2 cases), change in nodal stage (1 case), and change in tumour size or T stage (1 case). The single discordant case rated as having medium clinical impact by both medical oncologists included a change in both tumour size and lymphovascular invasion status. The 3 cases rated by both radiation oncologists as having medium clinical impact involved margin status. Table IV summarizes the potential therapeutic implications of discordant elements for the cases in which there was agreement between subspecialists regarding potential clinical impact.

## 4. DISCUSSION

In the mid-1990s, the primarily U.S.-based Association of Directors of Anatomic and Surgical Pathology recommended that review of outside pathology should be standard policy before patient treatment at a different institution 10. In practice, however, inter-institutional pathology consultation is variably applied 11,12. Suggested reasons for variability in the adoption of routine second-opinion pathology include workload constraints 5, financial costs 6, uncertainty about the value of reviews, and concerns about treatment delay 4.

Our institution had a policy of routine review upon referral in all cases of breast cancer patients with original pathology reported at an outside institution. Although the literature contains many studies of inter-institutional pathology consultations, our study is one of the few to examine breast cancer–specific inter-institutional pathology consultations and to assess the impact of discordant reporting on clinical decision-making.

Among 93 randomly selected inter-institutional pathology consultations for breast cancer at our institution, 10 (11%) had discordance with clinical impact as demonstrated by agreement within specialty by medical and radiation oncologist reviewers. Our results are in a range similar to that for discordance rates observed in other studies of inter-institutional pathology consultations.

In four large studies of inter-institutional pathology consultations in general pathology, rates of major discordance ranged from 1.4% to 9.0% 3–6. The lower value is from a study of 6171 cases referred to the Johns Hopkins Hospital, which mandates second opinion for surgical pathology from outside hospitals 3. In that study, a changed diagnosis was defined as “a discordant diagnosis resulting in a major modification in therapy or prognosis.” Manion *et al.,* using a similar definition of major discordance, observed a 2.3% major discordance rate and a 9.0% minor disagreement rate for 5629 cases in Iowa City 6. A review performed at the only cancer centre in Taiwan observed major discordance in 6% of mandatory inter-institutional pathology consultations 5. Weir *et al.* at the University Health Network in Toronto observed a 6.8% discordance rate in 1000 randomly selected consultations 4. In both the Iowa City and Toronto studies, approximately half the discordant findings had clinical impact.

More recently, a study comparing pathology report discordance relating to patients with non-Hodgkin lymphoma in the National Comprehensive Cancer Network was reported. The authors observed discordant reports in 43 of 731 cases for an overall rate of 6%. Of the discordant cases, 35 (81%) may have had treatment altered as a result of pathology reclassification 7.

In three of the four general pathology studies, discordance rates for breast cancer cases are reported or can be elucidated separately. Kronz *et al.* at Johns Hopkins found 1.4% of breast cases to be discordant 3, with most discordances involving differentiation between *in situ* and invasive disease, and 1 case involving a change in nodal status. The Toronto group reported discordance in 4.1% of breast cases 4. In the Taiwanese series, 6.3% of breast cases were observed to be discordant, with most of those involving differentiation between *in situ* and invasive disease, or between histologic tumour types 5. In our study, inter-institutional pathology consultation revealed invasive components in 2 cases originally reported as ductal carcinoma *in situ*. Other reasons for case discordance with agreement on clinical impact included tumour size, hormone receptor status, nodal stage, margin status, and presence of lymphovascular invasion. No case in our study involved an incorrect diagnosis of a breast neoplasm.

Staradub *et al.* reported major changes in surgical therapy in 7.8% of cases resulting from second-opinion pathology review after a biopsy diagnosis of invasive or *in situ* breast carcinoma 13. Most of those cases involved discordance between *in situ* and invasive disease or a change in margin status. In 80% of cases in that study, there were also differences in pathology or prognostic factors between the original and the review opinions.

In a study of 149 breast cancer cases undergoing multidisciplinary case review in Michigan, pathology interpretation changed in 43 (29%), and surgical plans were altered in 13 (9%) based on pathology review alone 9. A similar study of 77 patients revealed a major discordance in 3 (4%) cases, all of which led to a change in therapy 8.

Our study differs from the investigations of changes in surgical management by our focus on the clinical effect of discordant pathology reports for recommendations regarding adjuvant systemic or radiation therapy for breast cancer. As a result, elements included in our study—such as changes in tumour size, hormone receptor status, and lymphovascular invasion—were not specifically noted by the other studies. Interestingly, despite differences in methodology and definitions of clinical impact, the rate of discordance with clinical impact in our study was consistent with rates observed in previous studies.

Our results have certain limitations. We designed our study to require clinicians to make an explicit statement about the impact of the pathology consultation in the context of each individual patient scenario. Providing a summary of the original and consultation reports, together with information about patient age and type of surgery, aimed to simulate the setting in which clinical decision-making occurs; however, this approach cannot be assumed to accurately reflect an actual clinical decision reached at the oncology consultation. Our methodology focused on individual oncologist assessment of clinical impact, which may be subject to considerable variability based on expertise or clinical focus. Indeed, in 22 of 93 consultations, medical oncologists disagreed on independently performed ratings of clinical impact, and in 13 of 93, radiation oncologists disagreed.

The criteria for designation of clinical impact were specified and operationalized to maximize consistency. However, the clinical judgment of the individual reviewer may have resulted in differences in the interpretation of the relative clinical importance of the discordant reports. For example, one medical oncologist more often commented on additional surgical management; the other focused predominantly on changes in systemic therapy.

The inclusion of “different emphasis on a treatment modality” in the criteria for medium impact may also have increased variability. Some disagreement is not unexpected and likely represents areas of clinical uncertainty and variation in practice patterns. We did not allow for consensus discussion to resolve disagreements, as may occur during formal multidisciplinary tumour rounds. Our methodology may therefore overestimate the degree of clinical disagreement that might otherwise be mediated by consensus opinion, and it cannot be generalized to multidisciplinary tumour boards as exist in most major oncology centres, including our own.

Some discordant elements in our study were identified as a result of re-cutting of tissue blocks during the pathology consultation. The examination of additional sections is common during pathology consultations and may yield additional information even when adequate tissue sampling occurred during the initial gross specimen evaluation. Thus, in theory, the findings of the consultation report may reflect evaluation of additional slides rather than discordance in interpretation. We were unable to determine the extent to which additional sectioning occurred in our study because we collected data from pathology reports, but did not perform a retrospective pathology review as part of our protocol.

The second pathology opinion, often performed at a referral centre by an expert in the disease site of interest, is often assumed to be correct. However, in a small percentage of cases, studies that obtained clinical follow-up have observed that the original interpretation was indeed correct or that neither the initial nor the second opinion was correct 3,5,6. It has been suggested that the true “gold standard” in assessment of the accuracy of a pathology report is the clinical course of the disease 6, but that course is usually altered by therapeutic interventions and is difficult to directly relate to specific pathologic factors.

It was encouraging to note that our study identified no case originally diagnosed as malignant that was reviewed as benign, and no misdiagnosis of a non-carcinomatous breast neoplasm such as lymphoma, sarcoma, or secondary neoplasm metastatic to the breast. It remains possible, however, that neoplasms incorrectly diagnosed as benign could be underrepresented, because most cases with an original diagnosis of benign breast disease would not be systematically referred to the QEII Health Sciences Centre and would not, therefore, have been included in our cohort of potentially eligible cases.

It was also reassuring to find only 2 discordant results involving hormone receptor status. Our study found only one estrogen and progesterone receptor–negative (er−/pr−) tumour that was subsequently reported as er+/pr+. In Nova Scotia, a few hospitals perform hormone receptor testing on site; the remainder of the testing is performed at the QEII Health Sciences Centre 14. To minimize the risk of false negatives, er−/pr− tumours diagnosed at hospitals performing on-site testing are routinely reviewed upon patient referral to the QEII for adjuvant therapy recommendations.

The consultations in our study occurred in 2004, which was before province-wide synoptic reporting for breast cancer was implemented in Nova Scotia. Although missing information was not a major factor in the 10 discordant consultations consistently rated as having clinical impact, reports that would be incomplete according to current synoptic reporting guidelines were not infrequent (data not shown). Whether the adoption of synoptic reporting has affected discordance in breast cancer pathology reporting in Nova Scotia is not known at this time, but synoptic reporting should—at least theoretically— result in fewer reports with missing information on key prognostic factors.

## 5. CONCLUSIONS

Of 93 randomly selected inter-institutional pathology consultations for breast cancer, 10 (11%) were found to have discordance with either high or medium clinical impact as determined by agreement on clinical impact ratings within specialty by medical and radiation oncologist reviewers. Many consultations had discordant elements that either had no clinical impact or for which reviewers disagreed on potential impact. These results further inform our breast site team on the practice and merits of inter-institutional pathology consultation for breast cancer in Nova Scotia. They will provide a basis for further research and quality assurance initiatives and will help to guide human resource requirements in pathology. These results may also stimulate other investigators to examine similar questions within the Canadian context.

## Figures and Tables

**TABLE I:** Data elements abstracted from the original and consultation pathology reports

Type of data	Elements abstracted
Referral information	Referring hospital
	Surgeon
	Original and consulting pathologists
	Patient age
	Type of surgery
	Dates of surgery and pathology reports
	Number of slides and blocks submitted
	Use of synoptic reporting format
Pathologic features of invasive carcinoma	Tumour size
	Histologic type
	Chest wall, skin, nipple involvement
	Distribution (multifocal, multicentric)
	Nottingham grade
	Lymphovascular invasion
	*In situ* component subtype and nuclear grade
	Extensive intraductal component status
	Resection margin status
	Distance to nearest margin
	If margin involved, specify site
	If margin involved, *in situ* or invasive at margin
	If margin involved, macroscopic or microscopic
	Type of nodal surgery
	Number of nodes resected and number involved
	Size of nodal metastases
	Presence of extranodal extension
	Hormone receptor status
	Human epidermal growth factor receptor (her2/*neu*) status
Pathologic features of *in situ* carcinoma	Size
	Extent (number of blocks involved)
	Histologic type and subtype
	Distribution (multifocal, multicentric)
	Resection margin status
	Distance to nearest margin
	If margin involved, specify site
	Type of nodal surgery (if applicable)
	Number of nodes resected and number involved
	Size of nodal metastases
	Presence of extranodal extension

**TABLE II:** Pre-specified criteria for discordant elements to be classified as having no clinical impact

Pathologic feature	Discordance with no clinical impact
Distance of tumour to closest margin	• Distance different, but 2 mm or more in both reports
	• Distance 2 mm or more in one report and not described in other report
*In situ* component	• Size of *in situ* component not described
	• Stated as not present in one report and not described in other report
Extracapsular nodal extension	• Stated as not present in one report and not described in other report
Extensive intraductal component	• Stated as negative in one report and not described in other report
Nipple, skin, chest wall	• Omitted in description if uninvolved
Hormone receptor status	• Not performed at referring hospital or pending at time of referral
her2/*neu*	• Not considered for discordance, because tested only at QEII Health Sciences Centre

**TABLE III:** Clinical–pathologic characteristics of included cases derived from 93 original surgical pathology reports

Characteristic	[*n* (%)]
Breast-conserving surgery	49 (53)
Modified radical mastectomy	44 (47)
Invasive carcinoma	81 (87)
*In situ* carcinoma	12 (13)
Hormone receptor status	
Negative	5 (5)
Positive^a^	32 (34)
Not determined or not described^b^	56 (60)
Nodal status	
Not involved	49 (53)
Involved	28 (30)
Not determined or described	16 (17)

a Includes estrogen receptor (er) +/progesterone receptor (pr) +, er+/pr−, and er−/pr+.

b Includes cases from hospitals that do not perform hormone receptor testing on site and cases in which hormone receptor testing was performed on site, but is still pending at time of referral.

**TABLE IV:** Clinical features of cases with agreement on clinical impact within oncology speciality^a^

Case	Age	Surgery	Pathology		Main discordant elements	Potential therapeutic implications
			Original	Consultation		
*High clinical impact*						
1	61	Breast-conserving surgery	Ductal carcinoma *in situ*	Ductal carcinoma *in situ* with **microinvasion**	Microinvasion	Further surgery
2	66	Modified radical mastectomy, axillary lymph node dissection	**Ductal carcinoma*in situ,*** 1.5 cm Nodes negative	**Invasive ductal carcinoma** T1aN0 Grade 2 lvi-negative	Invasion	Offer hormone therapy
3	55	Modified radical mastectomy, axillary lymph node dissection	Invasive ductal and invasive lobular carcinoma, **0.5 cm** **T1a**N0 Grade 2 lvi-negative	Invasive ductal and invasive lobular carcinoma, **1.3 cm** **T1c**N0 Grade 1 lvi-negative er+/pr+	Tumour stage	Offer systemic adjuvant therapy
4	71	Modified radical mastectomy, axillary lymph node dissection	Invasive ductal carcinoma T2N2 Grade 2 lvi-positive **er+/pr+**	Invasive ductal carcinoma T2N2 Grade 3 lvi-positive **er**−**/pr**−	Hormone receptor status	Do not offer hormone therapy
5	51	Breast-conserving surgery sentinel lymph node	Invasive ductal carcinoma T1a**N1mi** (1/3 micrometastasis) Grade 2 lvi-negative	Invasive ductal carcinoma T1a**N0(i+)** (2/3 nodes with isolated tumour cells) Grade 2 lvi-negative er+/pr+	Nodal stage	Do not offer chemotherapy; perform axillary lymsph node dissection
6	51	Breast-conserving surgery, axillary lymph node dissection	Invasive ductal carcinoma T1cN0 Grade 1 lvi-negative **er**−**/pr**−	Invasive ductal carcinoma T1cN0 Grade 1 lvi-negative **er+/pr+**	Hormone receptor status	Offer hormone therapy
*Medium clinical impact*						
7	56	Modified radical mastectomy, axillary lymph node dissection	Invasive ductal carcinoma, **2.0 cm** **T1c**N0 Grade 3 lvi**not described**	Invasive ductal carcinoma, **2.2 cm** **T2**N0 Grade 3 lvi**present** er−/pr−	Tumour size LVI status	More emphasis on chemotherapy
8	77	Breast-conserving surgery, mammary node	Invasive ductal carcinoma T2N0 **Margins negative (5 mm)** Grade 3 Cannot determine lvi	Invasive ductal carcinoma T2N0 **Margins negative (1 mm)** Grade 3 lvi present er+/pr+	Change in distance to nearest margin	Add radiotherapy boost
9	43	Completion mastectomy, axillary lymph node dissection	Invasive ductal carcinoma, size not clear N1 **Ductal carcinoma*in situ*6 mm from margin** Grade 3 lvi absent er+/pr+	Invasive ductal carcinoma T1cN1 **Ductal carcinoma*in situ*less than 1 mm from margin** Grade 3 lvi present er+/pr−	Change in distance to nearest margin	Add radiotherapy boost
10	60	Breast-conserving surgery, axillary lymph node dissection	Invasive ductal carcinoma with lobular features **T1b**N1 **Ductal carcinoma*in situ*less than 1 mm from margin** Grade 3	Invasive ductal and invasive lobular carcinoma **T1c**N1 ***In situ*and invasive carcinoma 2 mm from margin** Grade 3 lvi absent er+/pr+	Change in distance to nearest margin	Do not add radiotherapy boost

a Boldface type emphasizes areas of discordance.

lvi = lymphovascular invasion; er = estrogen receptor; pr = progesterone receptor.
